# Inter-species comparative antioxidant assay and HPTLC analysis of sakuranetin in the chloroform and ethanol extracts of aerial parts of *Rhus retinorrhoea* and *Rhus tripartita*

**DOI:** 10.1080/13880209.2017.1304428

**Published:** 2017-03-27

**Authors:** Perwez Alam, Mohammad Khalid Parvez, Ahmed Hassan Arbab, Nasir Ali Siddiqui, Mohammed Salem Al-Dosary, Adnan Jathlan Al-Rehaily, Sarfaraz Ahmed, Mohd Abul Kalam, Mohammad Shamim Ahmad

**Affiliations:** a Department of Pharmacognosy, College of Pharmacy, King Saud University, Riyadh, Kingdom of Saudi Arabia;; b Department of Pharmacognosy, College of Pharmacy, Omdurman Islamic University, Khartoum, Sudan;; c Nanomedicine Research Unit, Department of Pharmaceutics, College of Pharmacy, King Saud University, Riyadh, Kingdom of Saudi Arabia

**Keywords:** Anacardiaceae, flavanone, DPPH assay, β-carotene bleaching method, validated HPTLC

## Abstract

**Context:** Extensive research on *Rhus* (Anacardiaceae) shows their antioxidant potential, which warrants further evaluation of its other species.

**Objective:** To perform a comparative antioxidant assay on extracts of *R. retinorrhoea* and *R. tripartita*, including sakuranetin quantification by a validated HPTLC method.

**Materials and methods:***In vitro* antioxidant assay was performed on chloroform and ethanol extracts of *R. retinorrhoea Steud. ex Oliv.* (RRCE and RREE) and *R. tripartita (Ucria) Grande* (RTCE and RTEE) by DPPH radical scavenging (at 31.25, 62.5, 125, 250 and 500 μg/mL concentrations) and β-carotene-linoleic acid bleaching methods at 500 μg/mL concentration. Densitometric HPTLC method was developed and validated using toluene: ethyl acetate: methanol (8:2:0.2; v/v/v) as mobile phase, executed on glass-backed silica gel F_254_ plate and scanned at 292 nm.

**Results:** Antioxidant activity of *Rhus* extracts tested by the two methods (DPPH/BCB) was found in order of RTEE > RREE > RTCE > RRCE with IC_50_ 118.67/256.26, 315.75/82.35, 827.92/380.0 and 443.69/292.75, respectively. Scanning of the HPTLC plate provided an intense peak of sakuranetin at R_f_ = 0.59. The estimated sakuranetin content in the dry weight of the extracts was highest in RREE (27.95 μg/mg) followed by RRCE (25.22 μg/mg), RTEE (0.487 μg/mg) and RTCE (0.0 μg/mg). Presence of sakuranetin in RREE, RRCE and RTEE supported the highest antioxidant property of the two *Rhus* species. Nonetheless, low sakuratenin in *R. tripartita* indicated the presence of other bioactive constituents responsible for synergistic antioxidant activity.

**Conclusion:** The developed HPTLC method therefore guarantees its application in quality control of commercialized herbal drugs and formulations containing sakuranetin.

## Introduction

Exploring novel and effective naturally derived antioxidants of nutritional values for human health has been a major goal for herbalists. The genus, *Rhus* (Anacardiaceae) commonly known as ‘sumac’ is comprised of 250 flowering plant species distributed in the temperate and tropical regions, worldwide (USDA 2007). Traditionally, several species of *Rhus* have been used for their medicinal purposes by indigenous cultures (Van Wyk & Wink 2004). Of these, *Rhus glabra* L. (smooth sumac) (Anacardiaceae) has been used in the treatment of bacterial infection like syphilis, gonorrhea, dysentery, and gangrene by North American people (Erichsen-Brown 1989). Other species, *Rhus coriaria L.* (tanner’s sumac) is commonly used as spice and wound healing herb in the Mediterranean and Middle East regions (Sezik et al. 1991). Moreover, maximum research dedicated to *Rhus* extracts has shown their antioxidant potential that warrants for further evaluations of its other species and commercialization.

The ethanol extract of *Rhus verniciflua Stokes* was reported to exhibit strong antioxidant properties against reactive oxygen species wherein its aqueous fraction protected against thymocyte apoptosis, attributed to antioxidant flavonoid contents (butein, quercitin, fustin and sulfuretin) (Lee et al. 2001). In addition, fustin and sulfuretin were found in *Rhus copallina*, *Rhus typhina* (Young 1976) and *R. glabra* (Keppler 1957; Yasue & Kato 1957) which had suggested their potential antioxidant properties. The ethanol extract of *R. verniciflua* wood was found superior to BHA and α-tocopherol in its antioxidative action (Park et al. 2002). The alcoholic extract of fruits and leaves of *R. coriaria* is used in stabilization of peanut oil due to its high antioxidant properties (Ozcan 2003a, 2003b). *Rhus tripartita* is mainly found in North Africa (Tackholm 1974) and widely used in food and traditional medicine (Wu et al. 2013). It has been extensively used in the treatment of several ailments including diarrhea, inflammatory diseases, diabetes, sexual disease, fever, pain and various cancers (Lev & Amar 2002; Giancarlo et al. 2006; Kossah et al. 2010; Lee et al. 2010). Furthermore, several flavonoid phytoconstituents with mild antimalarial activity have been also reported in *Rhus retinorrhoea* (Ahmed et al. 2001).

**Figure 1.**
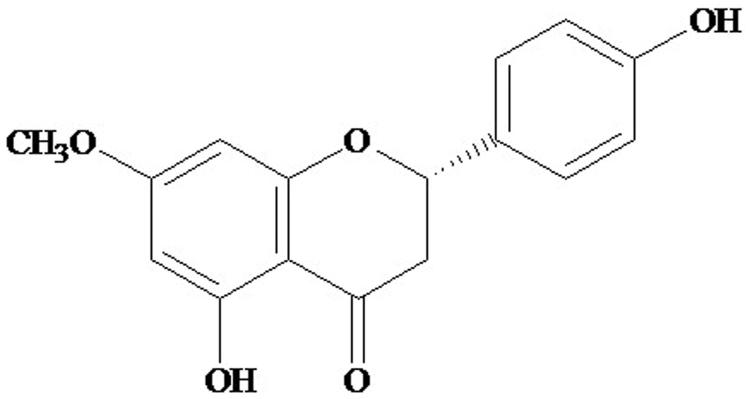
Chemical structure of flavonoid sakuranetin.

Flavonoids are the phenolic compounds of plant origin, and exhibit antioxidant, antimicrobial, antiallergenic, antiviral, anti-inflammatory and photoreceptor activities. The antioxidant property of flavonoid is attributed to its ability to reduce the formation of free radicals and promotion of free radical scavenging (Pier-Giorgio 2000). Of these, sakuranetin [(+/−) 7-*O*-methylnaringenin] is an important flavanone (Figure 1) (Jung et al. 2005) that has been identified in several other genera like, *Oryza sativa* L. (Grasses) (Rakwal et al. 1996), *Boesenbergia pandurata* (Roxb.) Schltr. (Zingiberaceae) (Tuchinda et al. 2002), *Eriodictyon californicum* (Hook. & Arn.) Torr. (Hydrophylloideae) (Liu et al. 1992) and different Piper species (Orjala et al. 1994; Moreira et al. 2000). Sakuranetin has been found to possess antioxidant (Soarse et al. 2005), chemopreventive (Charles et al. 2014), antinoceptive, and anti-inflammatory properties (Zhang et al. 2006; Cruz et al. 2016).

**Figure 2.**
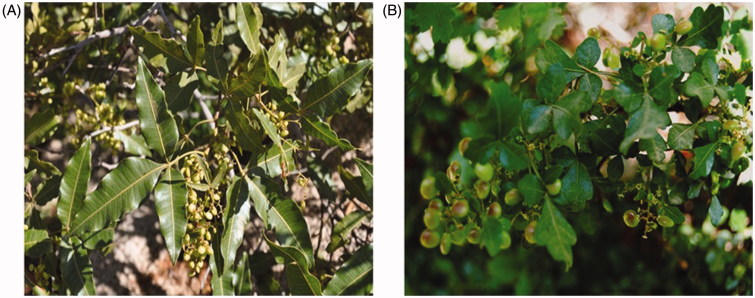
Pictures of *Rhus* species under study. (A) Picture of *R. retinorhoea*. (B) Picture of *R. tripartite*.

Based on this background information indicating promising antioxidative potential of *Rhus* extracts with few applied examples, we argue that this genus may offer a promising natural source of commercial antioxidants. Therefore, we performed in this study a comparative antioxidant assay of chloroform and ethanol extracts of aerial parts of *Rhus retinorrhoea* (Figure 2(A)) and *Rhus tripartita* (Figure 2(B)) along with the estimation of sakuranetin by a validated HPTLC method.

## Materials and methods

### Plant material

The aerial parts of *R. retinorrhoea* (Voucher number-15371) were collected in March 2009 and *R. tripartita* (Voucher number-15807) were collected in March, 2012 from northern part of Saudi Arabia. These samples were authenticated by Dr. Mohammed Yusuf (Field taxonomist, Pharmacognosy Department) and specimens were deposited at the college herbarium.

### Plant material extraction by using ultrasonic method

The aerial parts of *R. retinorrhoea* (RR) and *R. tripartita* (RT) were dried in air, pulverized and passed through a 0.75 mm sieve. The extraction process was carried out in an ultrasonic cleaner Transsonic-460/H (*ELMA,* Germany). The powdered material of RR (5.0 g) and RT (5.0 g) were extracted by ultrasonication (frequency 20 kHz, power 100 W) using ethanol (95%) and chloroform, separately. The chloroform and ethanol extracts (CE and EE, respectively) were centrifuged at 5000 rpm for 20 min, filtered through Whatman filter paper No. 1. Therefore, the obtained extracts (CE and EE) of the two *Rhus* species (RT and RR) were concentrated and dried under reduced pressure using rotary evaporator (R-210, BUCHI). The estimated yields (w/w) of RRCE, RREE, RTCE and RTEE were 4.52, 8.75, 3.86 and 5.62%, respectively.

### Apparatus and reagents

Sakuranetin (Biomarker; purity: ≥95%), rutin (antioxidant standard; purity: ≥94%), Tween___40, 2,2-diphenyl-1-picrylhydrazyl (DPPH), β-carotene, linoleic acid were purchased from Sigma Aldrich (USA). In addition, the AR grade toluene, ethyl acetate, chloroform, ethanol were procured from BDH (UK) and HPLC grade methanol were procured form Merck (Germany) where, glass-backed silica gel 60F_254_ plate was also procured for the HPTLC analysis. Furthermore, CAMAG Automatic TLC Sampler-4 (Switzerland) was used to apply the sakuranetine and the *Rhus* extracts (RRCE, RREE, RTCE and RTEE), band wise to the chromatographic plates and development of the plate was carried out in automatic development chamber (ADC2) (Switzerland). The developed HPTLC plates were then scanned and documented by CATS 4 (CAMAG) and TLC Reprostar 3 (CAMAG), respectively.

### Antioxidant assay

#### 1,1-Diphenyl-2-picrylhydrazyl (DPPH) radical scavenging assay

Antioxidant activities of RRCE, RREE, RTCE and RTEE were evaluated quantitatively by free-radical scavenging ability against DPPH as per the previously described method (Lee et al. 2013) with minor alteration to suite 96-well microtitre plate format. To sum up, 100 μL of different concentrations (31.25, 62.5, 125, 250 and 500 μg/mL) of each extract was mixed with 40 μL of DPPH (0.2 mM in methanol) in wells of a 96-well microtitre plate. Moreover, appropriate control was prepared using the solvent only in addition to the same amount of DPPH reagent to get rid of any inherent solvent effect. Rutin was used as standard. After 30 min incubation in dark at 25 °C, the decrease in absorbance (Abs) was measured at λ= 517 nm using microtitre plate reader. The test was carried out in triplicate. The radical scavenging activity was calculated from the equation:

$$\%\;\text{Radical scavenging activity}=\left[\text{1}-\left({\text{Abs}}_{\text{sample}}/{\text{Abs}}_{\text{control}}\right)\right]\times \text{1}00$$

#### β-Carotene-linoleic acid bleaching assay

The antioxidant activities of RRCE, RREE, RTCE and RTEE were evaluated by using the β-carotene bleaching method (Miller 1971) with minor modifications for working with 96 well plate. Briefly, 0.25 mg β-carotene was dissolved in 0.5 ml of chloroform and added to flasks containing 12.5 μg of linoleic acid and 100 mg of Tween-40. The chloroform was evaporated at 43 °C using speed vacuum concentrator (Savant, Thermo Electron Co.). The resultant mixture was immediately diluted to 25 mL with distilled water and shaken vigorously for 2–3 min to form an emulsion. A 150 μL aliquot of the emulsion was added to the wells of a 96-well microtitre plate containing 50 μL of each plant extract or rutin at 500 μg/mL. In addition, a control containing solvent instead of extract was also prepared. The plate was incubated at 50 °C for 2 h. Absorbance was taken at 470 nm at 30 min intervals using microplate spectrophotometer (BioRad Laboratories Inc., Hercules, CA). The test was carried out in triplicate. Then, antioxidant activity was expressed as % inhibition of lipid peroxidation using the formula:

$$\%\;\text{Inhibition}=\left[\left({\text{Abs}}_{\text{sam12}0}-{\text{ Abs}}_{\text{cont12}0}\right)/\left({\text{Abs}}_{\text{con}0}-{\text{ Abs}}_{\text{cont12}0}\right)\right]\times \text{1}00$$

where Abs_sam120_ and Abs_cont120_ are the absorbance of the sample and control, respectively, at time 120 min, and Abs_con0_ is the absorbance of the control at time 0 min.

#### HPTLC instrumentation and conditions

The HPTLC analysis of sakuranetin in RRCE, RREE, RTCE and RTEE was carried out on NP-HPTLC plates (20 × 10 cm) where the band size of each track was 6 mm wide and 9.4 mm apart. The marker and samples were applied on HPTLC plate (160 nL/sec), and developed in pre-saturated twin-trough glass chamber (20 × 10 cm) at 25 ± 2 °C under 60 ± 5% humidity. The developed HPTLC plate was dried and quantitatively analyzed at 292 nm wavelength in absorbance mode.

#### Preparation of standard stock solutions

The fresh stock solution of sakuranetin (1 mg/mL) was prepared in methanol and further diluted to furnish different concentrations (10-180 μg/mL) in the same solvent. All samples were applied (10 μL, each) through microliter syringe attached with the applicator on the HPTLC plate to provide the linearity range of 100–1800 ng/band.

#### Validation of method

Validation of the proposed HPTLC method was performed according to the ICH guidelines, for the determination of linearity range, limit of detection (LOD), limit of quantification (LOQ), precision, recovery as accuracy and robustness (ICH guidelines, 2005).

### Statistical analysis

The statistical analysis was carried out by one-way analysis of variance (ANOVA) followed by Dunnet’s test for the estimation of total variation in a set of data. Results were expressed as mean ± SD. *p* < 0.01, which was considered significant.

## Results

### Antioxidant activity (*in vitro*)

**Figure 3.**
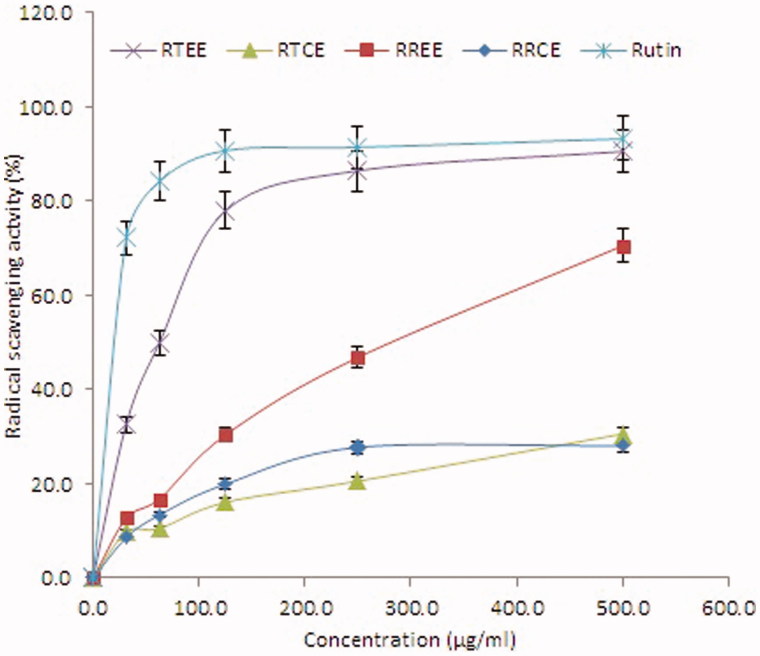
DPPH radical scavenging activity of different concentrations (31.25–500 μg/ml) of RRCE, RREE, RTCE and RTEE. Values are means of three experiments.

**Figure 4.**
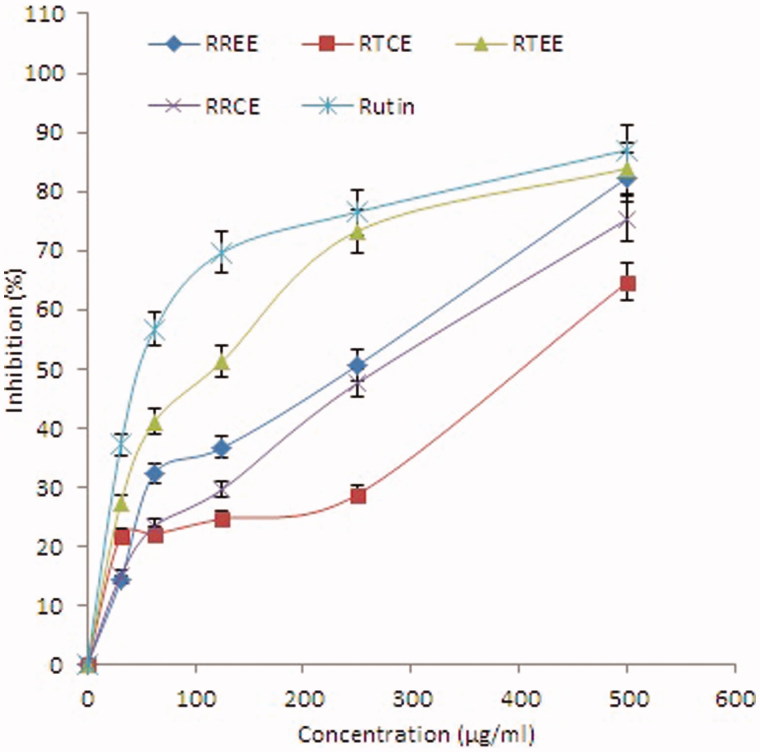
Antioxidant activity of RRCE, RREE, RTCE and RTEE in comparison to the standard antioxidant (rutin) assayed by the β-carotene bleaching method showing percentage of inhibition of lipid peroxidation by different concentrations (31.25–500 μg/ml) of the extracts. Values are means of three experiments.

Free radical scavenging activities (DPPH) of RRCE, RREE, RTCE and RTEE were tested for the first time and the results are represented in Figure 3. The IC_50_ values for RRCE, RREE, RTCE and RTEE in DPPH assay were found to be 443.69, 315.75, 827.92 and 118.67 μg/mL, respectively, whereas in the case of β-carotene-linoleic acid bleaching assay they were found to be 292.75, 82.35, 380.00 and 256.26 μg/mL, respectively. At the concentration of 500 μg/mL, the maximal antioxidant activity was found in the ethanol extracts, RTEE (90.6%) and RREE (70.5%). However, the chloroform extracts (RTCE and RRCE) of the two plants had no significant difference. The radical scavenging activity of the RTEE was comparable to that of the positive control rutin (93.3%). In accordance to the findings of β-carotene-linoleic acid bleaching assay, RTEE and RREE showed higher antioxidant potential than RTCE and RRCE (Figure 4). Taken together, the ethanol extract of *R. tripartita* had higher antioxidant potential than the other extracts of *R. tripartita* and *R. retinorrhoea*.

### HPTLC method development and validation

**Figure 5.**
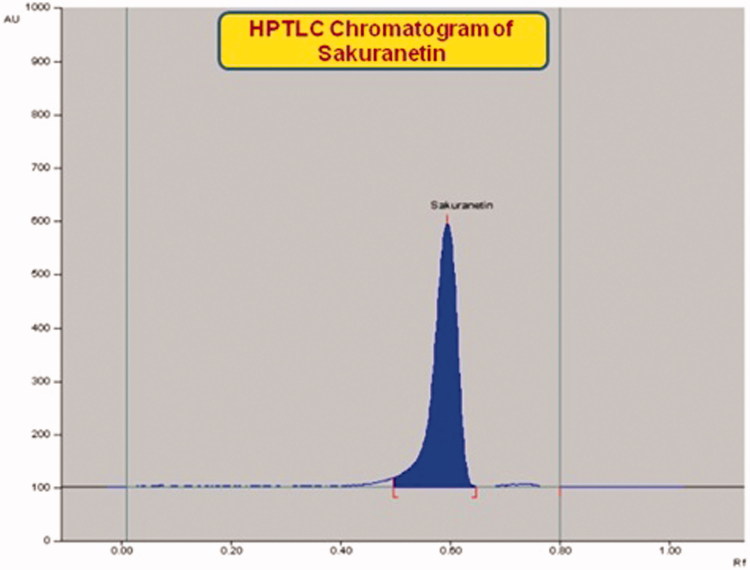
Chromatogram of standard sakuranetin (R_f_ = 0.59; 400 ng/spot) at 292 nm.

**Figure 6.**
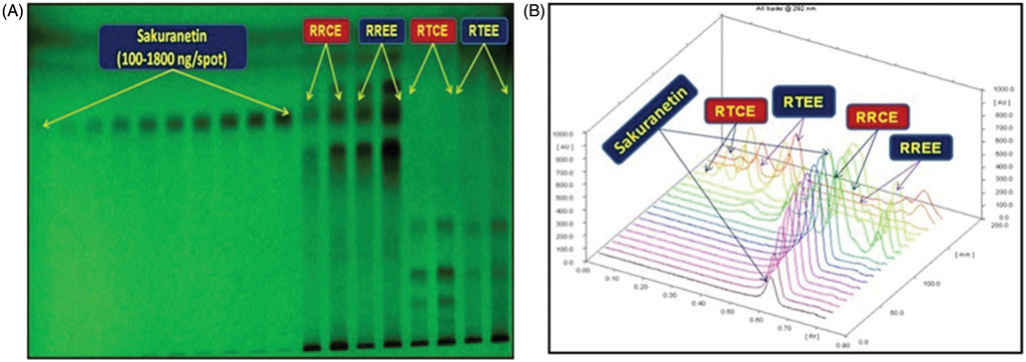
Quantification of sakuranetin in the extracts of two *Rhus* spp. by HPTLC. (A) Pictogram of developed HPTLC plate at 254 nm [mobile phase: Toluene: EA: Methanol, (8:2:0.2, v/v/v)]; (B) 3-D display of all tracks at 292 nm.

**Figure 7.**
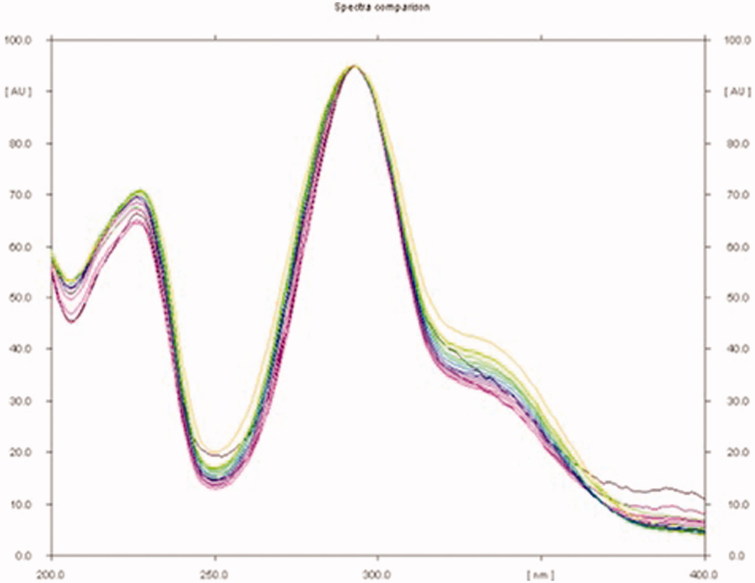
Spectral comparison of all tracks at 292 nm.

The mobile phase used in HPTLC analysis was selected by trying several compositions of different solvents. Of these, combination of toluene, ethyl acetate and methanol in the ratio of 8:2:0.2 (v/v/v) under chamber saturation condition was found to be the best mobile phase for the development and quantitative analysis of sakuranetin. The developed method was able to provide an intense, compact and sharp peak of sakuranetin at R_f_ 0.59 ± 0.003 (Figure 5). This method clearly separated the biomarker sakuranetin and different constituents of RRCE, RREE, RTCE and RTEE (Figure 6). The optimized saturation time and mobile phase volume for saturation were found to be 20 min and 20 ml, respectively. The identities of the bands were confirmed by overlaying their spectra along with sakuranetin (Figure 7). The developed methods was therefore, found to be selective with high resolution baseline. The regression equation and correlation co-efficient (*r*^2^) for sakuranetin were found to be *Y* = 15.382*x* + 5325.43 and 0.9979 ± 0.0010 in the linearity range 100–1800 ng/spot. The limit of detection (LOD) and limit of quantification (LOQ) for sakuranetin were found to be 27.56 and 83.53 ng/band, respectively (Table 1). The recoveries as accuracy study for the proposed method was recorded (Table 2). The recovery (%), RSD (%) and SEM for sakuranetin were 98.65–99.58%, 0.229–0.298% and 0.186–0.550, respectively. The intra-day and inter-day precision for the proposed method was recorded (Table 3). The %RSD for intra-day and inter-day precisions (*n* = 6) were found to be 0.185–0.293% and 0.173–0.279%, respectively, which showed the good precision of the proposed method. The robustness data of sakuranetin were reported in the Table 4. The low SD, % RSD and SEM values indicated that the method was robust.

**Table 1.** R_f_, Linear regression data for the calibration curve of sakuranetin (*n* = 6).

Parameters	Sakuranetin
Linearity range (ng/spot)	100–1800
Regression equation	*Y* = 15.382*x* + 5325.43
Correlation (*r*^2^) coefficient	0.9979 ± 0.0010
Slope ± SD	15.382 ± 0.128
Intercept ± SD	5325.43 ± 19.952
Standard error of slope	0.052
Standard error of intercept	8.143
R_f_	0.59 ± 0.003
LOD	27.56 ng/band
LOQ	83.53 ng/band

**Table 2.** Recovery as accuracy studies of the proposed HPTLC method (*n* = 6).

Percent (%) of Sakuranetin added to analyte	Theoretical concentration of Sakuranetin (ng/ml)	Concentration of Sakuranetin found (ng/mL) *±* SD	%RSD	SEM	% Recovery
0	200	199.17 ± 0.456	0.229	0.186	99.58
50	300	295.94 ± 0.883	0.298	0.360	98.65
100	400	396.74 ± 1.053	0.265	0.429	99.18
150	500	494.74 ± 1.348	0.272	0.550	98.94

**Table 3.** Precision of the proposed HPTLC method (*n* = 6).

	Intra-day precision			Inter-day precision		
Conc. of Sakuranetin (ng/band)	Average Conc. found ± SD	%RSD	SEM	Average Conc. found ± SD	%RSD	SEM
400	398.15 ± 0.978	0.245	0.399	395.95 ± 0.913	0.231	0.372
600	598.67 ± 1.11	0.185	0.453	596.03 ± 1.03	0.173	0.421
800	797.42 ± 2.344	0.293	0.956	795.73 ± 2.228	0.279	0.909

**Table 4.** Robustness of the proposed HPTLC method (*n* = 6).

	Sakuranetin (300 ng/band)		
Optimization condition	SD	%RSD	SEM
Mobile phase composition; (Toluene: ethyl acetate: methanol)			
8:2:0.2	0.916	0.309	0.374
7.8:2.2:0.2	0.886	0.301	0.362
8.2:1.8:0.2	0.943	0.322	0.385
Mobile phase volume (for saturation)			
18 ml	0.916	0.309	0.374
20 ml	0.876	0.297	0.357
22 ml	0.943	0.318	0.385
Duration of saturation			
10 min	0.872	0.295	0.871
20 min	0.918	0.312	0.918
30 min	0.976	0.332	0.975

### HPTLC analysis of sakuranetin

**Figure 8.**
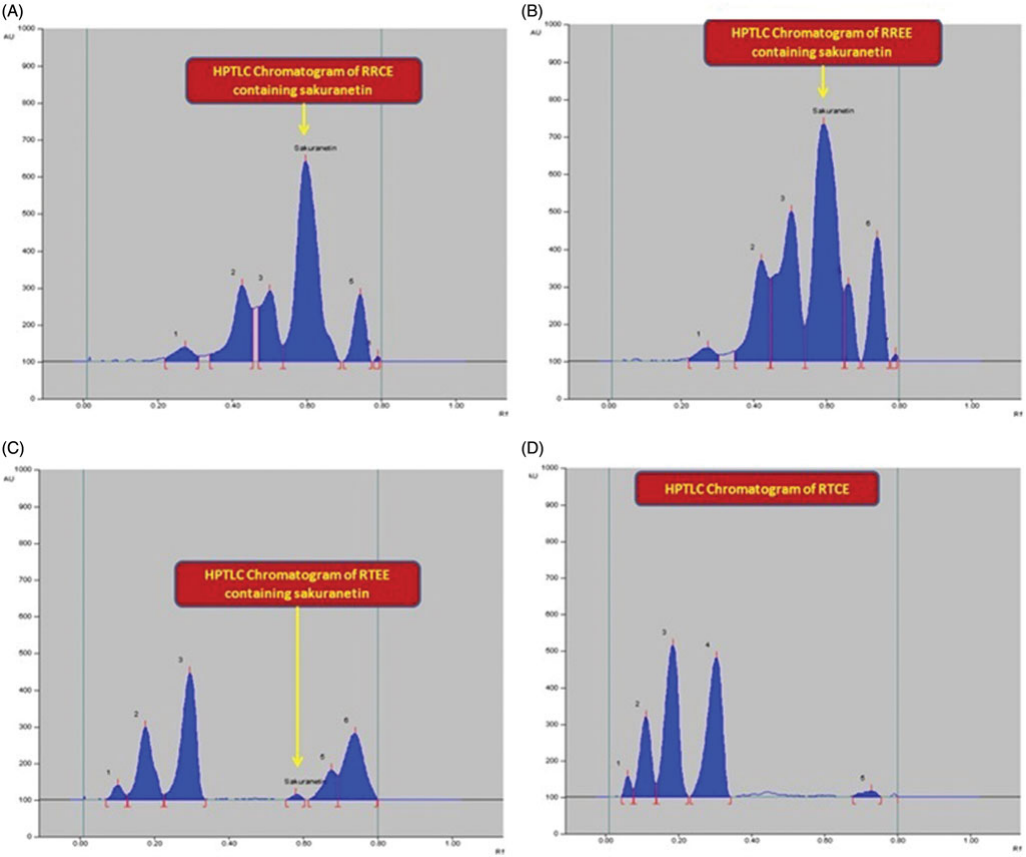
Chromatogram of sakuranetin estimation in the extracts of *Rhus* spp. at 292 nm [mobile phase: Toluene: EA: Methanol, (8:2:0.2, v/v/v)]. (A) *R. retinorhoea* chloroform extract (RRCE; spot 4, R_f_ = 0.59); (B) *R. retinorhoea* ethanol extract (RREE; spot 4, R_f_ = 0.59); (C) *R. tripartita* ethanol extract (RTEE; spot 4, R_f_ = 0.59); (D) *R. tripartita* chloroform extract (RTCE; Sakuranetin is absent).

The proposed HPTLC method was used for quantitative analysis of sakuranetin in the *Rhus* extracts RRCE, RREE, RTEE and RTCE (Figure 8). By applying this method, the estimated quantity of sakuranetin was 27.95 μg/mg for RREE (Figure 8(B)), 25.22 μg/mg for RRCE (Figure 8(A)) and 0.487 μg/mg for RTEE (Figure 8(C)) of the dried weight of extracts. However, sakuranetin was not detected in RTCE (Figure 8(D)). This maiden report thus, demonstrated the development of an economical, precise, accurate and simple HPTLC method for quantitative analysis of sakuranetin in different extracts of *R. retinorrhoea* and *R. tripartita*.

## Discussion

It is commonly recommended to perform different assay methods for evaluation of antioxidant activity (Alam et al. 2013). In DPPH assay, an antioxidant molecule provides an electron to DPPH which results in decay of its purple color. The measured decrease in the absorbance therefore, correlates with the free radical scavenging potential of the test sample. This represents one of the most common methods used to measure an extract’s ability to scavenge free radical. In addition, this is a favoured method because of its simplicity and compatibility with hydrophilic, lipophilic, pH, temperature and light sensitive nature of antioxidant samples (Kedare & Singh 2011). On the other hand, in β*-*carotene bleaching method, the linoleic acid radical formed by the loss of a hydrogen atom from one of its diallylic methylene groups attacks unsaturated β-carotene molecules. As a result, β-carotene is oxidized and subsequently the system loses its chromophore and characteristic orange colour. In our *in vitro* assays, the more polar ethanol extract (RTEE and RREE) contains more phenolic compounds, therefore exhibited the highest antioxidant activity in comparison to the chloroform fraction of both plants. The available literature also revealed the presence of several phytoconstituents, like flavonoids, biflavonoids and isobiflavonoids in *R. tripartita* (Mahjoub et al. 2004, 2005; Alimi et al. 2013) which supports our findings of high-antioxidant property of RTEE. It was found that the sakuranetin content was more in RREE than RTEE. Sakuranetin has been proved to be a good antioxidant. However, its presence in high quantity in RREE and low quantity in RTEE is not only responsible for good antioxidant activity of RREE and RTEE, which reflected that there were several other flavonoids and phenolic compounds present in RREE and RTEE. The cumulative affect along with sakuranetin imparts excellent antioxidant property to RRTE and RREE. Our findings also approved the previous antioxidant finding of *R. tripartita* (Abd El-Salam 2015). The antioxidant activity of phenolic phytoconstituents is attributed to their redox properties, which make them to act as reducing agents and hydrogen donors.

According to published reports, sakuranetin has been analyzed in different plants by several techniques, like nuclear magnetic resonance (NMR) (Jerz et al. 2005); IR & UV (Zhang et al. 2006), Mass spectrometry (Danelutte et al. 2003); HPLC & LC–MS (Jung et al. 2005), and EI-MS (Ogawa et al. 2007). However, there is no validated HPTLC method reported so far, for the quality control of sakranetin in herbal formulations or drugs. Recently, HPTLC has been extensively used in quality control of herbal drugs because of its various unique features, like inexpensive, high sample throughput, and requirement of very less cleaning solvent (Alam et al. 2014). In individual capacity, HPTLC is a reliable analytical tool for identification of herbs and their constituents, purity and stability testing of their preparations and analysis of uniformity, including those of animal extracts, drugs and excipients. Therefore, it has been widely used in the standardization and quality control of formulated products viz. pharmaceuticals, cosmetics, herbal and nutritional supplements (Alajmi et al. 2013). The present validated HPTLC method developed for the estimation of sakuranetin in two *Rhus* species would be beneficial in helping and facilitating the quality control of herbal drugs.

## Conclusion

The finding of high degree of antioxidant property of alcoholic extracts of *R. retinorrhoea* and *R. tripartita* warrants further evaluation of the two species in the various chronic diseases caused by free radical-induced oxidative damages. Our developed HPTLC method may be further applied for the quality control of herbal drugs or formulations containing sakuranetin, including the study of degradation kinetics.
